# Whole-transcriptome sequencing revealed the ceRNA regulatory network of stable knockdown of the CD8A gene in chicken T lymphocytes

**DOI:** 10.1016/j.psj.2025.106318

**Published:** 2025-12-19

**Authors:** Yanli Du, Ru Zhang, Meiquan Li, Xiao Wang, Hongyan Zhang, Bo Zhang, Bo Liao, Kun Wang, Xiannian Zi, Teng Huang, Changrong Ge, Jieyu Ma, Ke Li, Aiguo Xin

**Affiliations:** a College of Agronomy and Life Sciences, Kunming University, Kunming, Yunnan 650200, China; b Yunnan Academy of Animal Husbandry and Veterinary Sciences, Institute of Poultry Science, Kunming, Yunnan 650000, China; c Yunnan Provincial Key Laboratory of Animal Nutrition and Feed, Yunnan Agricultural University, Kunming, Yunnan 650201, China

**Keywords:** CD8A, Interference, T lymphocytes, Whole-transcriptome analysis, ceRNA network

## Abstract

CD8 subunit alpha (**CD8A**) is an important gene in immunity and is involved in the functional regulation of T lymphocytes. Previous studies have confirmed that interfering with CD8A expression inhibits the proliferation and induces the apoptosis of T lymphocytes. However, the specific regulatory mechanism of CD8A in chicken T lymphocytes remains unreported. In this study, whole-transcriptome RNA sequencing analyses of chicken T lymphocytes with interference of CD8A were performed. The whole-transcriptome RNA sequencing results revealed 465 differentially expressed genes (**DEGs**), 108 differentially expressed miRNAs (**DEMs**), 679 differentially expressed lncRNAs (**DELs**), and 18 differentially expressed circRNAs (**DECs**). The competitive endogenous RNA (ceRNA) regulatory network analysis indicated that interference with CD8A may regulate “interleukin-8 receptor binding”, “positive regulation of the cellular defense response”, “glycosaminoglycan biosynthesis-keratan sulfate”, “glycosphingolipid biosynthesis-ganglio series”, etc., thereby regulating the development of chicken T lymphocytes and immunity. Moreover, we extracted eight genes that were significantly enriched in the GO and KEGG analyses, including MITF, ARID5B, IL8L2, RUNX1, JAK1, MKNK2, TIPARP, and ST3GAL1, which are considered to be immune genes that are regulated by miR-1306-5p, miR-24-3p_R+1, miR-2478_L-1_1ss2TG, miR-106a-5p, and miR-17-5p. These miRNAs are competitively combined with other lncRNAs, including MSTRG.3287.1, MSTRG.851.3, MSTRG.5709.2, MSTRG.8606.10, MSTRG.8606.9, MSTRG.6504.2, and MSTRG.2145.4, etc., via circRNAs, including cirRNA145 and cirRNA147. Therefore, CD8A may affect T-cell development and thereby influence the immunity of chickens through these ceRNA regulatory networks and signaling pathways.

## Introduction

**Fig. 1:**
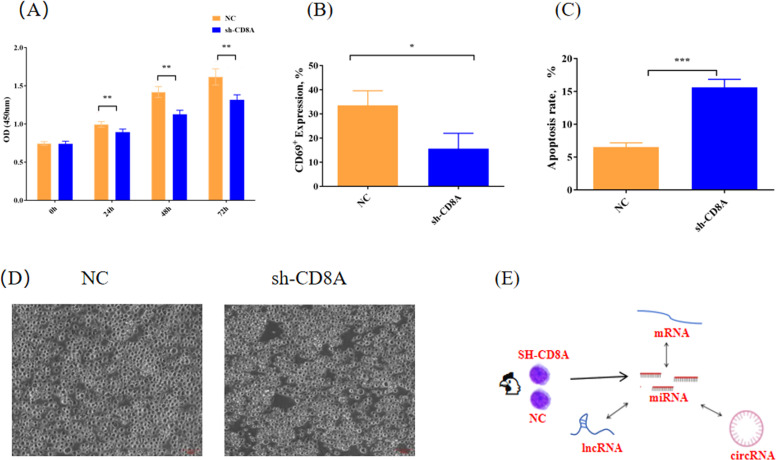
Effects of CD8A on the proliferation, apoptosis, and activation of T lymphocytes. Effects of CD8A on (A) the proliferative capacity of T lymphocytes, (B) the apoptosis of T lymphocytes, and (C) the activation of T lymphocytes were determined. (D) The morphology of chicken T lymphocytes in the NC and sh-CD8A groups was determined. (E) Schematic illustration of the experimental strategy. *, **, and *** represents *P* < 0.05, 0.01, and 0.001, respectively. sh-CD8A represents T lymphocytes with interference of CD8A, and NC represents T lymphocytes with no-load plasmid.

The CD8 molecule, a surface marker of T lymphocytes, binds to MHC class I molecules on antigen-presenting cells (APCs) that capture antigens, thereby increasing the interaction between T cells and APCs while also participating in the transmission of biological signals upon antigen stimulation to enable cytotoxic T lymphocytes (**CTL**) to recognize and eliminate antigens and infected cells (Dumontet et al., 2015). The CD8 molecule consists of heterodimers of CD8α and CD8β, which mainly exert an auxiliary effect on T-cell receptors (Miceli et al., 1993). The function of CD8 molecules is focused on the α chain, whereas that of CD8β is limited by the lack of the cysteine motif necessary for binding to lymphocyte kinase (**Lck**) (Xu et al., 2011). The α chain plays a key role in the immune response, whereas the β chain only acts as a scaffold during lymphocyte signaling (Wang et al., 2015). CD8α (**CD8A**) is highly expressed in the thymus and spleen of geese (Zhao et al., 2013). In mice, CD8A was found to bind to non-classical MHC class I thymoleukemia antigens (TL) independently of TCR, forming a novel mechanism for regulating T cells through CD8A-MHC Class I (Leishman et al. 2001). In pigs, single-nucleotide polymorphisms (**SNPs**) of CD8A are significantly associated with T lymphocyte subsets (Wang et al., 2015). CD8A can be a valuable and quantifiable prognostic indicator for the immunotherapeutic response and immune cell infiltration ratio (Ahmadzadeh et al., 2009). CD8A serves as a predictive biomarker for the prognosis and immunotherapy of bladder cancer (Zheng et al., 2022a). A study identified a haplotype (p.Gly111Ser) mutation in the CD8A gene in the Spanish Gypsy population; this mutation led to a shortage of CD8+ effector T cells (Xu et al., 2014a) and consequently affected immune function. CD8A is closely associated with the inflamed profile of T cells (Niu et al., 2022). In addition, in CD8A knockout mice, it was found that CD8A can also bind to PILRα and interact to maintain the quiescence of CD8+ T cells (Zheng et al., 2022b). Studies have also demonstrated the immunomodulatory function of CD8A in the intestinal subepithelial T cells of mice, which act as a barrier against intestinal epithelial tissue (Hayday et al., 2001). These results indicate that CD8A plays an important role in regulating immunity and is closely related to T cells. Our previous study revealed that interfering with CD8A expression inhibited the proliferation, differentiation, and activation of T lymphocytes and induced their apoptosis (Fig. 1A, B, and C) (Du et al., 2025). Additionally, the gene network regulating CD8A is not fully understood. Thus, an analysis of the function and related network regulation measures of CD8A, which is present on the surface of T lymphocytes, is important for gaining a deeper understanding of immunity in chickens.

Many studies have demonstrated that some non-coding RNAs, including lncRNAs, miRNAs, and circRNAs, perform regulatory functions by establishing intricate and precise post-transcriptional regulatory networks (Ma et al., 2018; Wang et al., 2020). Whole-transcriptome sequencing technologies, which offer a high-resolution snapshot of the transcriptomic landscape, have been extensively used to elucidate the molecular regulatory mechanism (Shen et al., 2016; Xu et al., 2016); for example, the researcher has performed whole transcriptome sequencing in 279 acute lymphoblastic leukemia (ALL) patients (B-cell: n = 211; T-cell: n = 68) to assess the accuracy of whole transcriptome sequencing, and found that whole transcriptome sequencing can perform precise genetic classification for most B Cell Precursor (BCP)-ALL patients (Walter et al., 2021). In addition, whole-transcriptome sequencing has been performed on the T lymphocytes, transcripts of interest have been identified (Jivanjee et al., 2022). However, studies on whole-transcriptome sequencing analysis of CD8A gene knockdown in chicken T lymphocytes have not been reported.

In this study, we analyzed the expression patterns of lncRNAs, circRNAs, miRNAs, and mRNAs to elucidate their roles in the knockdown of the CD8A gene in chicken T lymphocytes (Fig. 1D and E). Additionally, we conducted predictive analyses of ceRNA regulatory networks, including interactions among mRNAs, miRNAs, lncRNAs, and circRNAs. These findings revealed the intricate molecular regulatory network as a target of CD8A regulation in chicken T lymphocytes. Our results provided new insights into the role of CD8A in chickens.

## Materials and methods

### Ethics statement and experimental animals

The animal experimental procedures were approved and guided by the Kunming University Animal Care and Use Committee (approval ID: kmu2024042).

### Animals and sample collection

As described in our previous report (Du et al., 2025), we constructed a vector that interferes with the expression of the CD8A gene and transfected it into T lymphocytes to knock down CD8A. The chicken T lymphocytes were isolated from the thymus tissue and cultured. The chickens used in this study were 14-day-old AA broilers obtained from Yunnan Agricultural University. The group infected with viruses containing no load was denoted the NC group, whereas the group infected with pADV-U6-shRNA was denoted the shRNA-CD8A group. Samples from the NC and shRNA-CD8A groups were collected and quickly placed in liquid nitrogen, followed by storage at -80°C until further use.

### RNA extraction, library construction, and sequencing

Total RNA was extracted from T lymphocytes transfected with recombinant adenovirus (three samples each from the NC, sh-CD8A) using TRIzol reagent (Thermofisher, 15596018) following the manufacturer’s procedure. The quantity of RNA was detected using a Bioanalyzer 2100 system (Agilent, CA, USA). RNA purity was determined using an RNA 6000 Nano LabChip Kit (Agilent, CA, USA). For mRNA, circRNA, and lncRNA sequencing, 5 µg of total RNA was used to deplete rRNA with a Ribo-Zero Gold rRNA Removal Kit (Illumina, San Diego, USA). The remaining RNA was fragmented into short fragments using divalent cations at a high temperature. The final cDNA libraries generated 300 bp (±50 bp) reads using the NEBNext® Ultra^TM^ RNA Library Prep Kit for Illumina® (NEB, USA) and underwent high-throughput sequencing on the Illumina^TM^ NovaSeq 6000 platform; finally, 2 × 150 bp paired-end sequencing reads were generated.

To construct the small miRNA library, TruSeq Small RNA Sample Prep Kits (Illumina, San Diego, USA) were used to construct a miRNA library according to the manufacturer’s recommendations. Next, 3 µg of total RNA was added to 30 and 50 adapter-ligated RNA for the miRNA library. Finally, cDNA was generated and sequenced on an Illumina HiSeq 2000/2500 (LC-BIO, Hangzhou, China) to generate single-ended reads 50 bp in length.

### Alignment and assembly of RNA-seq data

To obtain clean data, raw RNA-seq reads were trimmed by Cutadapt (v1.9), and then FastQC was used to evaluate the quality of the original reads, including removing reads that contained adapters and low-quality reads. The clean reads generated were mapped to the chicken reference genome (ftp://ftp.ensembl.org/pub/release-107/fasta/gallus_gallus/dna/) using HISAT2 (v2.2.1). For circRNAs, the reads were aligned to the chicken reference genome using TopHat2 (v2.0.4). For the reads that were not aligned, TopHat2 was also used for supplementary genomic alignment. SAMtools (v1.1.0) was used to convert the files from SAM files to BAM files. The transcripts were assembled using StringTie (v2.1.6).

### Identification of lncRNAs, circRNAs, and miRNAs

Identification of lncRNAs: After assembling reads, transcripts shorter than 200 bp and those with known mRNAs, lncRNA overlaps were screened out. Long non-coding RNAs were subsequently predicted from the remaining transcripts. Next, CPC (version 0.9-r2) and CNCI (v 2) were used to predict lncRNA transcripts longer than 200 bp, and all transcripts with a CNCI score < 0 and a CPC score < 0.5 were retained and considered to be lncRNAs. For novel transcript identification, transcripts were reconstructed using Stringtie software (v2.1.6) and HISAT2 software (v2.2.1) to identify new genes and new splicing variants of known genes. To identify new transcripts, the gffcompare software (v0.9.8) was used to align all reconstructed transcripts to the reference genomes and classify them into 12 classes.

Identification of circRNAs: We used a method similar to lncRNA analysis to process circRNA sequencing data, including filtering and genomic alignment. CIRCExplorer2 (v2.2.6) and CIRI (v2.0.2) were used to assemble the mapped reads to circular RNAs de novo. Then, back-spliced junction reads were identified in unmapped reads by TopHatFusion, and CIRCExplorer2 or CIRI was used to identify back-spliced junction reads within unmapped reads.

Identification of miRNAs: ACGT101-miR (v4.2) removes adapter dimers, junk, low complexity, common RNA families (rRNA, tRNA, snRNA, and snoRNA), and repeat sequences. Known and novel miRNA sequences between 18 and 26 bp in length were located in precursors through miRBase 22.1. The names prefixed with “miRs” represent mature miRNAs, whereas those prefixed with “PC” represent newly discovered miRNAs.

### Analysis of DEGs/DELs/DECs/DEMs and functional enrichment analysis

***DEGs, DECs, DELs***, and ***DEMs*** were identified using the DESeq2 software, with |log2FC| ≥ 1 and *P* < 0.05 selected. We subsequently visualized these genes and generated heatmaps and volcano maps using the ggplot2 package in R. The ***DEMs*** with *P* < 0.05 were identified.

### Screening potential target genes of DELs/DECs/DEMs and functional enrichment analysis

For the lncRNAs, coding genes 100 kb upstream and downstream were selected by a Python script as the cis-target genes of the lncRNAs. The target genes of the ***DELs*** were subjected to enrichment analysis using GO and KEGG. All results were considered to be statistically significant at *P* < 0.05.

*The* ***DECs*** were predicted as miRNA (miRBase, http://www.mirbase.org) targets using TargetScan (v5.0) and miRanda (v3.3a) with TargetScan_score ≥ 50 and miRanda_Energy < –10. Differentially expressed circRNA host coding genes were subjected to enrichment analysis using GO and KEGG. The results were considered to be statistically significant at *P* < 0.05. The target genes of the significant ***DEMs*** were predicted using TargetScan and miRanda. In TargetScan, target genes with context score percentiles < 50 were removed, and in miRanda, target genes with maximum free energy (Max Energy) > –10 were removed. The target genes of the DEMs were subjected to enrichment analysis using GO and KEGG. The results were considered to be statistically significant at *P* < 0.05.

### Prediction of the ceRNA regulatory network

To investigate the interactions among differentially expressed mRNAs **(DEmRNAs), *DELs, DECs, and DEMs***, we constructed a circRNA/lncRNA-miRNA-mRNA regulatory network based on the ceRNA hypothesis. TargetScan and miRanda were used to predict miRNA–mRNA, miRNA–circRNA, and miRNA–lncRNA pairs. Cytoscape (version 3.9.1) was used to visualize the circRNA/lncRNA-miRNA-mRNA interaction network.

### RT-qPCR validation of DEGs, DELs, DEMs, and DECs

To determine the accuracy of the mRNA sequencing results, three DEGs, three DELs, three DEMs, and three DECs were randomly selected based on the whole-transcriptome sequencing results, and qPCR validation was conducted using the same RNA samples used for RNA-seq, with three biological replicates.

The RNA isolated from the experimental samples was reverse-transcribed into cDNA using the SYBR PrimeScript PLUS RT-RNA PCR Kit and Mir-X miRNA First-Strand Synthesis Kit (Takara, Beijing, China). A SYBR Premix Ex Taq II kit (Takara, Beijing, China) was used to assess the expression levels of the DEGs. The primers used were designed using Primer Premier 5 software (Table S1). β-actin was used as the internal reference for mRNAs, lncRNAs, and circRNAs, and U6 served as the internal reference for miRNAs. Relative gene expression was calculated using the 2^-ΔΔCT^ method, where ΔCT was defined as the CT value of the gene minus the CT value of the internal reference.

## Results

### DEG expression following CD8A gene knockdown in chicken T lymphocytes

**Fig. 2:**
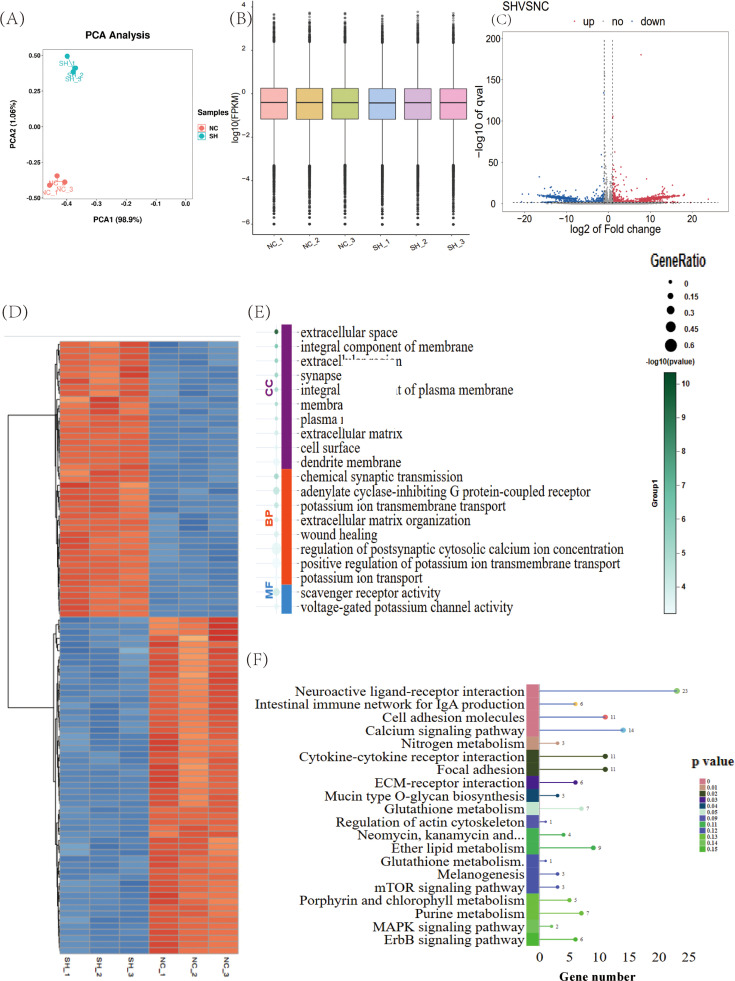
Expression of DEGs after the CD8A gene was knocked down in chicken T lymphocytes. (A) PCA plot of mRNA expression in all samples, with the SH group represented in cyan and the NC group in red. (B) Violin plot showing the expression levels of all detected genes across the six samples. Volcano plot (C) and heatmap (D) of the DEGs in the sh-CD8A vs. NC groups. Orange indicates upregulation, and blue indicates downregulation. (E) GO enrichment of DEGs. (F) KEGG enrichment analysis of DEGs, with the numbers indicating the magnitude of *P*-values, the top 20 pathways ranked by *P*-values are plotted.

To identify the RNAs involved in the development of T lymphocytes during CD8A knockdown, we performed whole-transcriptome sequencing analysis on T lymphocytes derived from the CD8A gene knockdown (SH-CD8A) and negative control (NC) groups. The results of the principal component analysis (**PCA**) revealed that the overall trend of mRNA distribution in the SH-CD8A and NC groups was separate in chicken T lymphocytes (Fig. 2A). The sequencing quality of the six samples remained relatively high (Fig. 2B). We subsequently identified 465 DEGs, among which 284 were upregulated and 181 were downregulated (Fig. 2C and Supplementary Table S2). The gene expression patterns in the samples reflect the credibility of the data. The results of the FPKM cluster analysis of the DEGs are shown in Fig. 2D. We subsequently conducted GO enrichment analysis on 465 DEGs, and 264 significant GO terms were identified, including “extracellular space”, “integral component of membrane”, “extracellular region”, etc. (Fig. 2E and Supplementary Table S3). KEGG pathway enrichment analysis was also performed on 465 DEGs, and 10 significant signaling pathways were enriched (*P* < 0.05). It mainly involves “neuroactive ligand-receptor interaction”, “the intestinal immune network for IgA production”, “cell adhesion molecules”, etc. (Fig. 2F and Supplementary Table S4).

### DELs expression following CD8A gene knockdown in chicken T lymphocytes

**Fig. 3:**
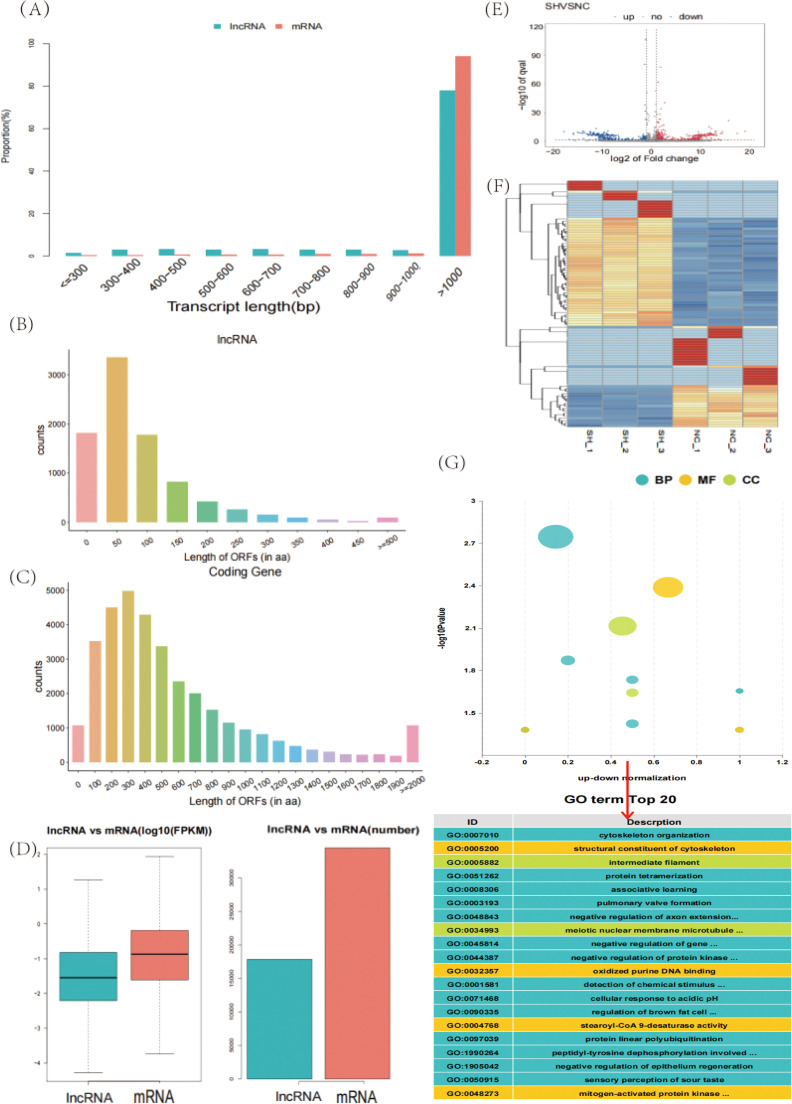
Expression analysis of lncRNAs was performed after the CD8A gene was knocked down in chicken T lymphocytes. (A) Length distribution of lncRNAs and mRNAs. (B) Distribution of ORF lengths of lncRNAs. (C) Distribution of the ORF lengths of mRNAs. (D) LncRNA and mRNA expression levels and lncRNA and mRNA number statistics. Volcano plot (E) and heatmap (F) of the DELs for sh-CD8A vs. NC. Orange indicates upregulation, and blue indicates downregulation. (G) GO enrichment of DELs; the top 20 pathways ranked by *P*-values are plotted. BP: biological process; CC: cellular component; MF: molecular function.

We analyzed the length distributions of the lncRNAs and mRNAs, and the results revealed that those with lengths greater than 1000 nt accounted for 77.87% and 99.23%, respectively (Fig. 3A). We also analyzed the open reading frame (**ORF**) lengths of the lncRNAs and mRNAs. The results revealed that 87.4% of the lncRNAs presented ORFs spanning 0–150 amino acids (Fig. 3B), whereas 67.2% of the mRNAs presented ORFs extending 100–600 amino acids (Fig. 3C). The mRNA expression level was greater than that of the lncRNAs, and the number of mRNAs was greater than that of the lncRNAs (Fig. 3D). We identified 679 DELs in the sh-CD8A and NC groups, including 429 upregulated and 250 downregulated genes (Fig. 3E and Supplementary Table S5). The gene expression patterns in the samples reflect the credibility of the data. The results of the FPKM cluster analysis of the DELs are shown in Fig. 2F. Next, GO analysis was conducted on the genes targeted by the DELs, and 32 significant GO terms were identified, which included “cytoskeleton organization”, “structural composition of cytoskeleton”, “intermediate filament”, etc. (Fig. 3G and Supplementary Table S6). KEGG pathway enrichment analysis was also performed, and no significant pathways were enriched.

### Expression of DECs following CD8A gene knockdown in chicken T lymphocytes

**Fig. 4:**
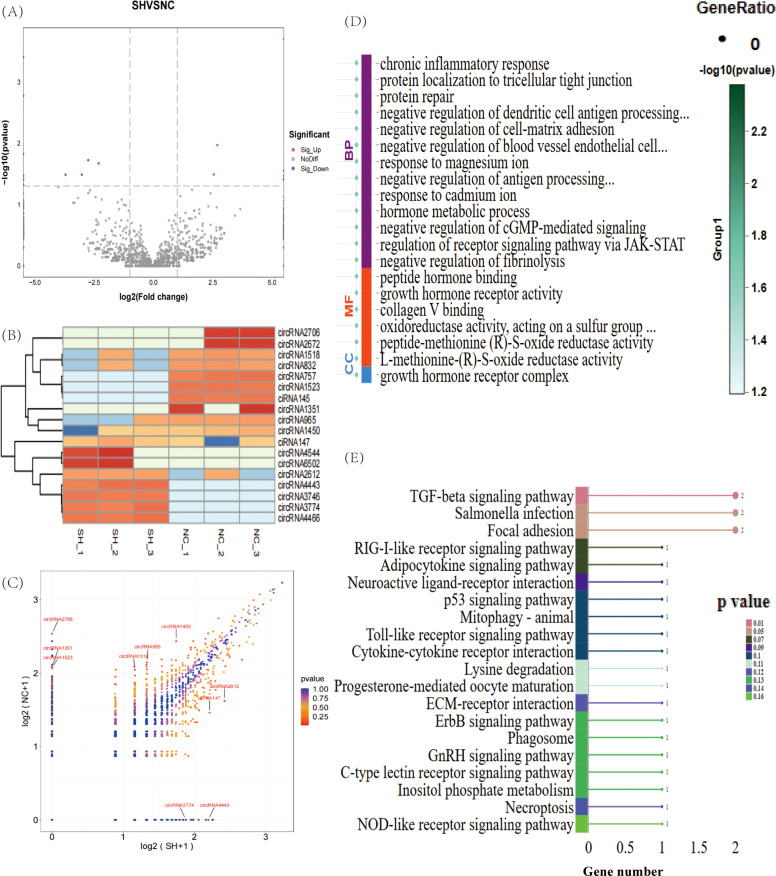
Expression analysis of circRNAs was performed after the CD8A gene was knocked down in chicken T lymphocytes. Volcano plot (A) and heatmap (B) of the DECs for sh-CD8A vs. NC. Orange indicates upregulation, and blue indicates downregulation. (C) Scatter plot of DECs. (D) GO enrichment of DECs. BP: biological process; CC: cellular component; MF: molecular function. (E) KEGG enrichment analysis of DECs; the top 20 pathways ranked by P-values are plotted.

We identified 18 DECs in the sh-CD8A and NC groups, including eight upregulated genes and 10 downregulated genes (Fig. 4A and Supplementary Table S7). The gene expression patterns in the samples reflect the credibility of the data. The results of the FPKM cluster analysis of DECs are shown in Fig. 4B. The scatter plot shows that the average expression levels of DECs varied greatly between the two groups (Fig. 4C). GO enrichment analysis was subsequently performed on the host genes of the DECs identified in the two groups, and 129 significant GO terms were identified. It mainly involved “chronic inflammatory response”, “protein localization to tricellular tight junction”, “peptide hormone binding”, etc. (Fig. 4D and Supplementary Table S8). KEGG pathway enrichment analysis revealed that three significant signaling pathways were co-enriched (*P* < 0.05), including “TGF-β signaling pathway”, “*Salmonella* infection”, and “focal adhesion” (Fig. 4E and Supplementary Table S9).

### Expression of DEMs following CD8A gene knockdown in chicken T lymphocytes

**Fig. 5:**
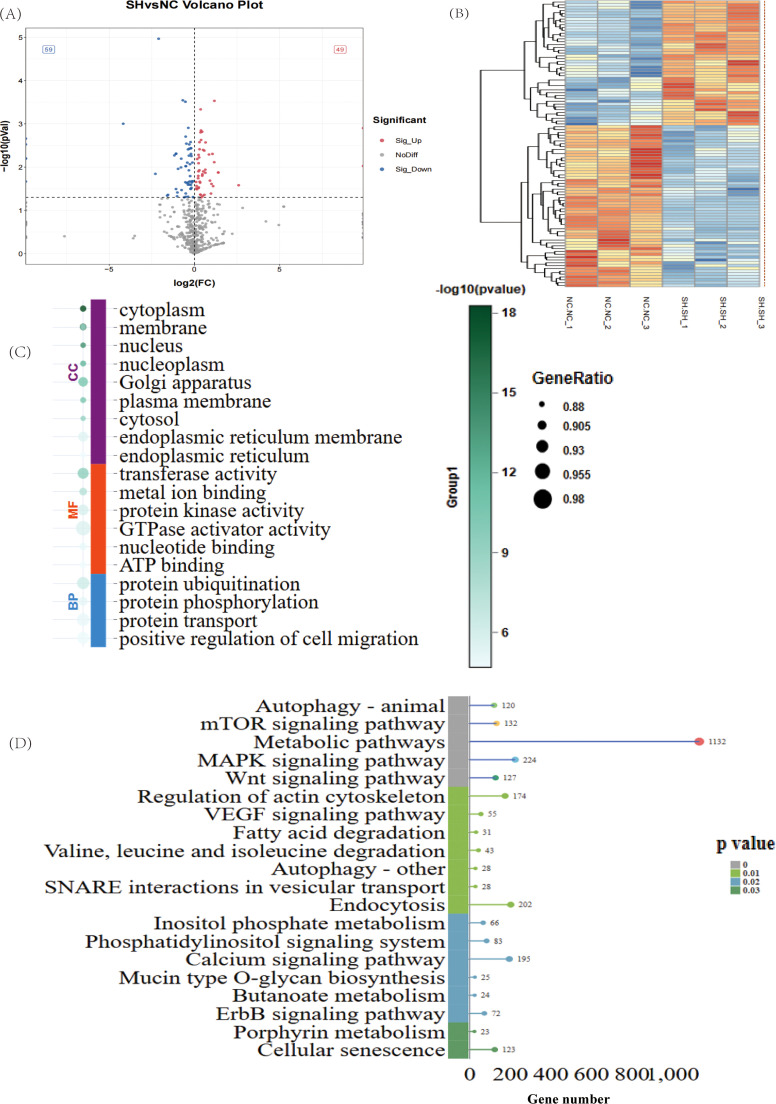
Expression analysis of miRNAs was performed after the CD8A gene was knocked down in chicken T lymphocytes. Volcano plot (A) and heatmap (B) depicting the DEMs or the sh-CD8A vs. NC. Orange indicates upregulation, and blue indicates downregulation. (C) GO enrichment analysis of DEMs. BP: biological process; CC: cellular component; MF: molecular function. (D) KEGG enrichment analysis of DEMs. The top 20 pathways ranked by *P*-values are plotted.

We identified 108 DEMs. Differential expression analysis revealed that 49 DEMs were significantly upregulated, whereas 59 DEMs were significantly downregulated (Fig. 5A-B and Supplementary Table S10). To further assess their potential functions, we conducted target gene prediction for all identified miRNAs (Supplementary Table S11). GO and KEGG analyses were subsequently conducted on the target genes of the DEMs. The results of the GO analysis revealed that the target genes of the DEMs were significantly enriched in 308 GO terms, such as “cytoplasm”, “membrane”, and “nucleus” (Fig. 5C and Supplementary Table S12). Moreover, the KEGG pathway results revealed that the target genes of the DEMs were significantly enriched in 27 pathways, including “autophagy-animal”, “mTOR signaling pathway”, and “metabolic pathways” (Fig. 5D and Supplementary Table S13).

### Construction of the lncRNA-miRNA-mRNA and circRNA-miRNA-mRNA ceRNA network

**Fig. 6:**
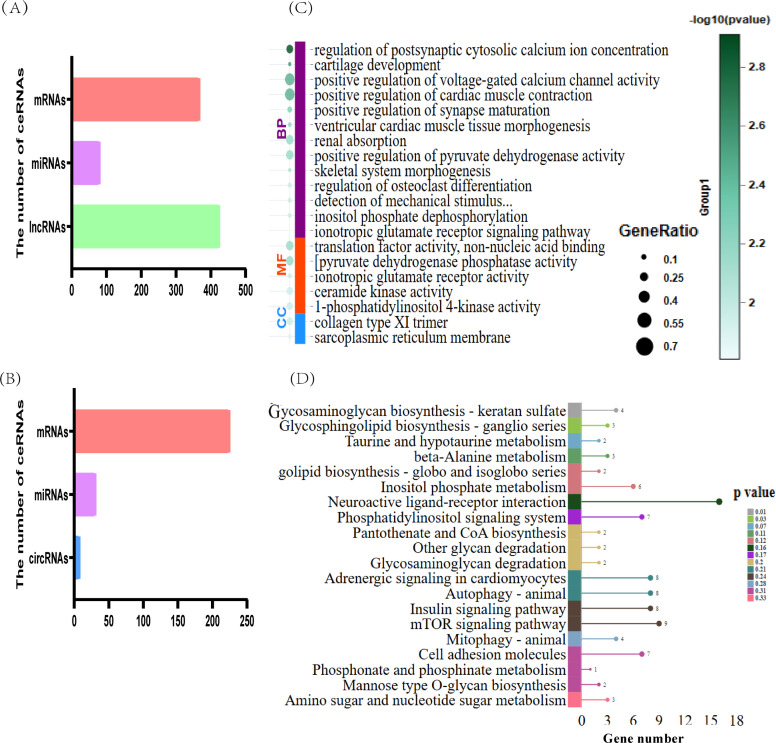
CeRNA prediction of all differentially expressed RNAs. (A) Number of mRNAs, miRNAs, and lncRNAs involved in lncRNA-miRNA-mRNA interactions. (B) Number of mRNAs, miRNAs, and circRNAs involved in circRNA-miRNA-mRNA interactions. (C) GO enrichment analysis of mRNAs involved in the ceRNA network. BP: biological process; CC: cellular component; MF: molecular function. (D) KEGG enrichment analysis of mRNAs involved in the ceRNA network. The top 20 pathways ranked by *P*-values are plotted.

**Fig. 7:**
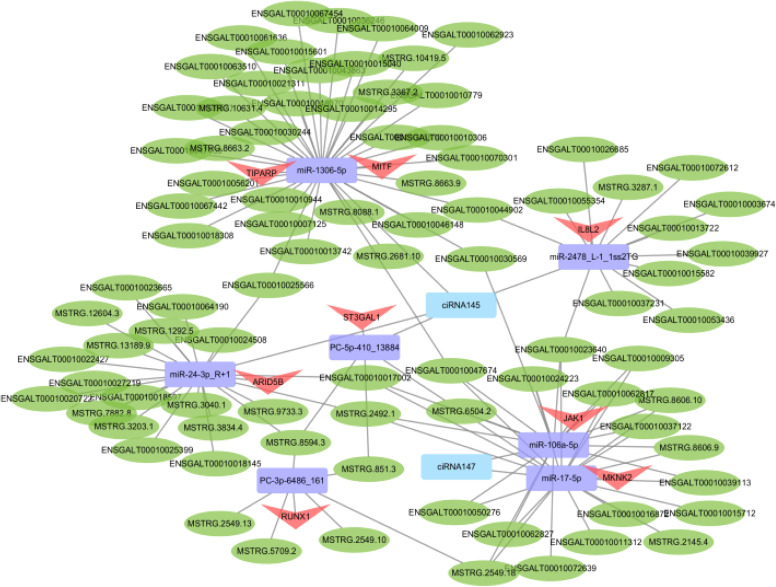
Illustration of the ceRNA regulatory network of circRNA/lncRNA-miRNA-mRNA interactions significantly enriched with target genes in T lymphocytes of chickens. The green circles represent lncRNAs, the purple rectangles represent miRNAs, the blue rectangles represent circRNAs, and the red triangles represent mRNAs.

By using TargetScan and miRanda for prediction, we established 1823 pairs of miRNA-mRNA interactions, 51 pairs of circRNA-miRNA interactions, and 787 pairs of lncRNA-miRNA interactions (Supplementary Tables S14–S16). With respect to the regulation of the lncRNA/circRNA-miRNA-mRNA networks, 655 circRNA-miRNA-mRNA interactions were obtained, including those involving seven circRNAs, 224 mRNAs, and 30 miRNAs (Fig. 6A and Supplementary Table S17). A total of 21,266 lncRNA-miRNA-mRNA interactions were obtained, including 423 lncRNAs, 366 mRNAs, and 79 miRNAs (Fig. 6B and Supplementary Table S18). Based on the mRNAs involved in the lncRNA/circRNA-miRNA-mRNA regulatory network, GO and KEGG enrichment analyses were performed. The GO enrichment analysis of the targeted mRNAs in these lncRNA/circRNA-miRNA-mRNAs revealed “memory B-cell differentiation”, “interleukin-8 receptor binding”, “positive regulation of the cellular defense response”, etc. (Fig. 6C and Supplementary Table S19). The KEGG enrichment analysis revealed that “glycosaminoglycan biosynthesis-keratan sulfate” and “glycosphingolipid biosynthesis-ganglio series” were enriched (Fig. 6D and Supplementary Table S20). To further identify the candidate ceRNA transcript-interaction relationships, we extracted eight genes that were significantly enriched in the GO and KEGG enrichment analyses. These genes are considered to be gene targets of immunity and include microphthalmia-associated transcription factor (MITF), AT-rich interaction domain 5B (ARID5B), interleukin 8 like 2 (IL8L2), RUNX family transcription factor 1 (RUNX1), Janus kinase 1 (JAK1), MAPK-interacting serine/threonine kinase 2 (MKNK2), TCDD-inducible poly(ADP-ribose) polymerase (TIPARP), and ST3 beta-galactoside alpha-2,3-sialyltransferase 1 (ST3GAL1). Therefore, we separately constructed ceRNA networks for these eight genes, involving lncRNA-miRNA-mRNA and circRNA-miRNA-mRNA interactions, and included seven miRNAs, 88 lncRNAs, two circRNAs, and 129 interaction relationships (Fig. 7).

#### RT-qPCR validation

**Fig. 8:**
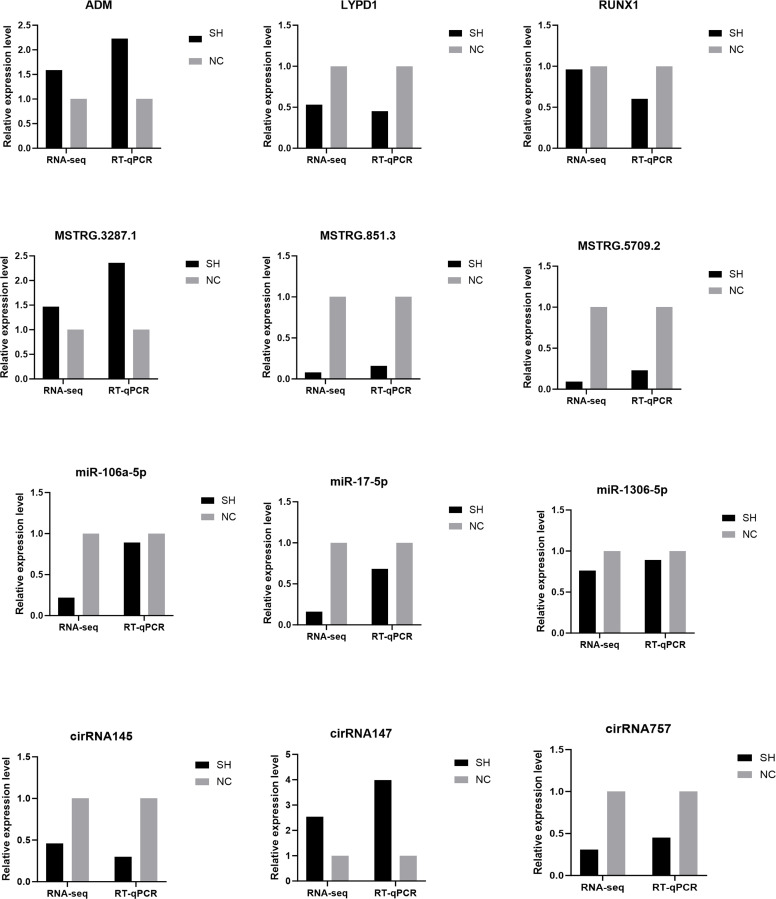
Validation of DEGs, lncRNAs, miRNAs, and circRNAs via reverse transcription-quantitative polymerase chain reaction (RT-qPCR).

Three randomly selected DE-mRNAs, DElncRNAs, DE-circRNAs, and DE-miRNAs were used to confirm the accuracy of the RNA-seq data (Fig. 8). The expression patterns of these genes, determined by RT-qPCR, were highly consistent with the results of the RNA sequencing (Fig. 8).

## Discussion

CD8A is essential for cell-mediated immune defense and T-cell development (Suetake et al., 2007). Our previous study revealed that interfering with CD8A expression inhibited the proliferation, differentiation, and activation of T lymphocytes and induced their apoptosis (Du et al., 2025). Therefore, CD8A helps regulate the development of T lymphocytes. The T lymphocytes of chickens are important immune cells and are regulated by various genes, transcription factors, and ncRNAs. However, no study has investigated the expression pattern and regulatory mechanism of CD8A in regulating the development of chicken T lymphocytes through lncRNAs, miRNAs, and circRNAs. Therefore, we analyzed the expression profiles of mRNAs, lncRNAs, circRNAs, and miRNAs during the stable knockdown of the CD8A gene in chicken T lymphocytes through whole-transcriptome analysis. Additionally, we constructed molecular regulatory networks to elucidate the interactions between ncRNAs and mRNAs.

In this study, 465 DEGs, 108 DEMs, 679 DELs, and 18 DECs were identified in the sh-CD8A vs. NC groups. The GO and KEGG enrichment analyses of the DEGs revealed that they were involved mainly in “neuroactive ligand-receptor interaction”, “institutional immune network for IgA production”, “cell adhesion molecules”, etc. The “neuroactive ligand-receptor interaction” pathways overlapped among the three comparisons (WT, sh-CD8A, and OE-CD8A). Another study reported that the “neuroactive ligand-receptor interaction” pathway is involved in the immune system during bacterial infection in teleost fish (Hou et al., 2023). DEGs such as adrenomedullin (ADM) and somatostatin receptor 2 (SSTR2) are involved in “neuroactive ligand-receptor interaction pathways”. ADM exerts immune protective effects by regulating the proliferation and differentiation of T cells and B cells (Kong et al., 2020; Simonetti et al., 2021). SSTR2 is a potential predictive biomarker for the response to immune checkpoint inhibitors (ICIs) (Wang et al., 2022). The “intestinal immune network for IgA production pathway” can protect the intestinal mucosa and maintain immune balance (Liu et al., 2024). DEGs such as interleukin 4 (IL4), interleukin 15 (IL15), and inducible T-cell costimulator ligand (ICOSLG) were involved in the “intestinal immune network for IgA production pathway”. IL4 plays a key role in the development of appropriate effector T-cell responses (Dhanda et al., 2013). Several studies have indicated that the application of IL-15 can increase the efficacy of CAR-T cells (Cieri et al., 2013; Xu et al., 2014b). ICOSLG mediates T-cell expansion (Iwata et al., 2020). “Cell adhesion molecules” at discrete stages of T-cell maturation participate in and regulate the complex processes of T-cell development (Pater et al., 1993). DEGs such as integrin subunit beta 2 (ITGB2) and ICOSLG were involved in “cell adhesion molecules”. ITGB2 may regulate the intercellular communication of cytotoxic CD8+ T cells (Lei et al., 2025). ICOSLG can regulate the immune microenvironment (Zhang et al., 2023). Thus, these significantly enriched signaling pathways may affect immunity in chickens. These results suggest that CD8A may regulate these significantly enriched signaling pathways and may affect the functions of T cells in chickens.

Based on the DEGs, DELs, DECs, and DEMs, we constructed circRNA-miRNA-mRNA and lncRNA-miRNA-mRNA regulatory networks for T cells. To investigate the potential functions of the ceRNA networks, we performed functional enrichment analysis for all differentially expressed mRNAs involved in these networks. The GO and KEGG analyses suggested that these genes participate extensively in signaling pathways associated with immunity, such as “memory B-cell differentiation”, “interleukin-8 receptor binding”, “positive regulation of the cellular defense response”, “glycosaminoglycan biosynthesis-keratan sulfate”, and “glycosphingolipid biosynthesis-ganglio series”. We identified eight genes that were significantly enriched in these enrichment analyses. These genes are considered potential gene targets of immunity and include MITF, ARID5B, IL8L2, RUNX1, JAK1, MKNK2, TIPARP, and ST3GAL1. MITF is necessary for T-cell maturation because it regulates the homing of dendritic cells (DCs) to the thymus (Karigane et al., 2022), thereby affecting the function of T cells. T-cell acute lymphoblastic leukemia (T-ALL) is a malignant disease characterized by abnormal proliferation of thymus T-cell precursors (Look, 1997). Some studies have reported that ARID5B promotes the occurrence of T-ALL (Leong et al., 2017). IL8L2 is a chemokine that plays a key role in the inflammatory and immune response of poultry (Song et al., 2020; Chao et al., 2025). RUNX1 is closely related to peripheral T-cell homeostasis (Mizutani et al., 2015). JAK1 promotes peripheral tolerance in autoimmunity through programmed cell death ligand 1 (PD-L1)-mediated regulatory T-cell induction (Vogel et al., 2022). Several studies have confirmed that MKNK2 is essential for the development and activation of T cells and regulates non-T-cell lineages to control the differentiation of Th1 and Th17 in vivo (Gorentla et al., 2013). TIPARP is a prognostic biomarker and potential immunotherapy target for male papillary thyroid carcinoma (Zhang et al., 2024a). ST3GAL1 can control chimeric antigen receptor (CAR)-T-cell migration to target tumor sites (Hong et al., 2023). Thus, CD8A may be a key regulator of these potential gene targets of immunity and may subsequently affect T-cell function and immunity in chickens. Finally, the eight notable DEmRNAs were used as the core to establish a ceRNA network model that included seven miRNAs, 88 lncRNAs, two circRNAs, and 129 interaction relationships. From the constructed ceRNA network, miR-1306-5p, miR-24-3p_R+1, miR-2478_L-1_1ss2TG, miR-106a-5p, and miR-17-5p, etc., were identified. Some of them were reported to affect immune function. A study found that gga-miR-1306-5p induced by *Salmonella enteritidis* or lipopolysaccharide regulates the immune response by inhibiting Toll-interacting protein, which activates the production of inflammatory cytokines (Sun et al., 2019). Studies have also shown that miR-106a-5p can upregulate various immunosuppressive/inflammatory molecules in peripheral blood mononuclear cells, such as CD38, and these molecules are involved in inflammatory responses, tumor necrosis factor-α signaling, and interleukin-6-JAK-STAT3 signaling (Mizuhara et al., 2023). Moreover, miR-106a-5p targets mitogen-activated protein kinase 2 (MAP3K2), regulates gene expression at the transcriptional level, and promotes the effective repair of intestinal barrier injury (Zhang et al., 2024b). Other studies have shown that miR-17-5p participates in regulatory T-cell-mediated immune escape of non-small cell lung cancer cells by targeting RUNX family transcription factor 3 (RUNX3) (Zheng et al., 2024). However, studies on miR-24-3p_R+1, miR-2478_L-1_1ss2TG, and the related functions of immunity and T cells have not been reported and need further assessment. Therefore, these several miRNAs may be involved in the CD8A-regulated T-cell network, thus becoming potential candidate miRNAs for regulating T-cell function and immunity.

## Conclusions

To summarize, in this study, we revealed that FMITF, ARID5B, IL8L2, RUNX1, JAK1, MKNK2, TIPARP, and ST3GAL1 are important immune target genes for the regulation of T cells by the chicken CD8A gene. These genes are regulated by miR-1306-5p, miR-24-3p_R+1, miR-2478_L-1_1ss2TG, miR-106a-5p, and miR-17-5p, which are competitively combined by lncRNAs, including MSTRG.3287.1, MSTRG.851.3, MSTRG.5709.2, MSTRG.8606.10, MSTRG.8606.9, MSTRG.6504.2, and MSTRG.2145.4, and by circRNAs, including cirRNA145 and cirRNA147. These findings provide new insights into how CD8A may affect the development of T-cells and thereby influence the immunity of chickens through the ceRNA regulatory network of circRNAs/lncRNAs-miRNAs-mRNAs.

## Acknowledgments

This work was financially supported by the Yunnan Basic Research Special Youth Project [202301AU070138, 202301AU070139], the Kunming University Talent Introduction Project [YJL23004], the Scientific Research Fund Project of Yunnan Provincial Department of Education [2023J0830], the Yunnan Province Basic Research Special General Project [202501AT070073], the NSFC [32260897], the Projects Funded of the Central Government to guide Local Scientific and Technological Development [202407AB110017], and the Innovation and Entrepreneurship Training Program for College Students [S202311393024].

Availability of Data and Materials: All data supporting our findings are included in the manuscript. The RNA sequencing data for this study can be found in the NCBI Sequence Read Archive (SRA) under Bioproject: PRJNA1378469 and PRJNA1177976.

## CRediT authorship contribution statement

**Yanli Du:** Writing – review & editing, Writing – original draft, Validation, Software, Methodology, Investigation, Funding acquisition, Formal analysis, Data curation, Conceptualization. **Ru Zhang:** Software. **Meiquan Li:** Investigation, Funding acquisition. **Xiao Wang:** Project administration, Methodology. **Hongyan Zhang:** Visualization, Validation. **Bo Zhang:** Resources. **Bo Liao:** Data curation. **Kun Wang:** Formal analysis. **Xiannian Zi:** Methodology. **Teng Huang:** Validation, Software, Resources. **Changrong Ge:** Methodology, Investigation, Funding acquisition, Data curation. **Jieyu Ma:** Resources. **Ke Li:** Resources. **Aiguo Xin:** Writing – original draft, Visualization, Validation, Supervision, Resources.

## Disclosures

The authors declare that they have no conflicts of interest.

## Appendix Supplementary materials

Image, application 1

Image, application 2

Image, application 3

Image, application 4

Image, application 5

Image, application 6

Image, application 7

Image, application 8

Image, application 9

Image, application 10

Image, application 11

Image, application 12

Image, application 13

Image, application 14

Image, application 15

Image, application 16

Image, application 17

Image, application 18

Image, application 19

Image, application 20
